# Protein glycosylation in cancers and its potential therapeutic applications in neuroblastoma

**DOI:** 10.1186/s13045-016-0334-6

**Published:** 2016-09-29

**Authors:** Wan-Ling Ho, Wen-Ming Hsu, Min-Chuan Huang, Kenji Kadomatsu, Akira Nakagawara

**Affiliations:** 1 School of Medicine, College of Medicine, Fu Jen Catholic University, New Taipei 24205, Taiwan; 2 Department of Pediatrics, Shin Kong Wu Ho-Su Memorial Hospital, Taipei, Taiwan; 3 Department of Pediatrics, National Taiwan University Hospital, Taipei, Taiwan; 4 Department of Surgery, National Taiwan University Hospital, Taipei, Taiwan; 5 Research Center for Developmental Biology and Regenerative Medicine, National Taiwan University, Taipei, Taiwan; 6 Graduate Institute of Anatomy and Cell Biology, College of Medicine, National Taiwan University, No. 1, Sec. 1, Jen-Ai Road, Taipei, 10051 Taiwan; 7 Department of Biochemistry, Nagoya University Graduate School of Medicine, Nagoya, Japan; 8 Saga Medical Center Koseikan, Saga, Japan

**Keywords:** Cancer, Glycan-based therapeutics, Glycosyltransferase, Lectin, Neuroblastoma, Protein glycosylation, Treatment

## Abstract

Glycosylation is the most complex post-translational modification of proteins. Altered glycans on the tumor- and host-cell surface and in the tumor microenvironment have been identified to mediate critical events in cancer pathogenesis and progression. Tumor-associated glycan changes comprise increased branching of *N*-glycans, higher density of *O*-glycans, generation of truncated versions of normal counterparts, and generation of unusual forms of terminal structures arising from sialylation and fucosylation. The functional role of tumor-associated glycans (Tn, sTn, T, and sLe^a/x^) is dependent on the interaction with lectins. Lectins are expressed on the surface of immune cells and endothelial cells or exist as extracellular matrix proteins and soluble adhesion molecules. Expression of tumor-associated glycans is involved in the dysregulation of glycogenes, which mainly comprise glycosyltransferases and glycosidases. Furthermore, genetic and epigenetic mechanisms on many glycogenes are associated with malignant transformation. With better understanding of all aspects of cancer-cell glycomics, many tumor-associated glycans have been utilized for diagnostic, prognostic, and therapeutic purposes. Glycan-based therapeutics has been applied to cancers from breast, lung, gastrointestinal system, melanomas, and lymphomas but rarely to neuroblastomas (NBs). The success of anti-disialoganglioside (GD2, a glycolipid antigen) antibodies sheds light on glycan-based therapies for NB and also suggests the possibility of protein glycosylation-based therapies for NB. This review summarizes our understanding of cancer glycobiology with a focus of how protein glycosylation and associated glycosyltransferases affect cellular behaviors and treatment outcome of various cancers, especially NB. Finally, we highlight potential applications of glycosylation in drug and cancer vaccine development for NB.

## Background

Glycosylation is the most complex post-translational modification of proteins and is involved in many physiological events, such as host-pathogen interaction, cell differentiation and trafficking, and intracellular and intercellular signaling. Tumor-associated glycan changes comprise increased branching of *N*-glycans, higher density of *O*-glycans, generation of truncated versions of normal counterparts (Tn, sTn, and T antigens), and generation of unusual forms of terminal structures with sialic acid and fucose (sLe^a^ and sLe^x^ epitopes), mainly caused by the genetic and epigenetic desregulation of glycogenes and the tumor microenvironment. Changes in oligosaccharide structures of glycoproteins or glycolipids are involved in cancer progression through the dysregulation of cell cycle, promotion of cell proliferation and growth, degradation of the extracellular matrix (ECM) and basement membranes, promotion of tumor dissemination and angiogenesis, and facilitation of immune evasion [1]. A massive potential for glycan diversity exists; however, a limited range of glycans are associated with invasion and metastatic potential in various tumors [1]. The endogenous animal lectins (glycan-binding proteins) participate in fundamental processes such as quality control of secreted proteins, host-pathogen recognition, cell adhesion, and motility. The interactions of lectins with tumor-associated glycans facilitate tumor progression in lung cancers, colon cancers, pancreatic carcinomas, melanomas, and neuroblastomas (NBs) [2–6].

NB is the most common extracranial solid tumor in children and the most common solid tumor of infancy, accounting for about 8–10 % of childhood cancers and for about 15 % of cancer deaths in children. The median age of children at diagnosis is 22 months, and 90 % of cases are diagnosed by 5 years of age. The annual incidence is estimated to be about 1/70,000 in children under the age of 15 [7]. There are ~150 new cases diagnosed each year in Japan and 30~40 new cases diagnosed each year in Taiwan according to the Japan Neuroblastoma Study Group and the Registry of Childhood Cancer Foundation of Taiwan, respectively [8, 9]. NB is a genetically and clinically heterogeneous cancer arising from embryonal sympathetic nervous system, exhibiting from spontaneous differentiation or regression with a favorable prognosis to highly undifferentiated tumors with rapid progression and very poor outcomes [7]. Although the overall prognosis of NB patients has improved remarkably with recent therapeutic advances, long-term survival of aggressive forms of NB remains poor even with intensive multimodal therapy [7]. The Children’s Oncology Group stratified patients into low-, intermediate-, or high-risk groups based on age at diagnosis, International Neuroblastoma Staging System (INSS) stage [10], tumor histology, DNA index (ploidy), and *MYCN* (V-myc myelocytomatosis viral-related oncogene) amplification status [11]. The osteomedullary recurrences from residual disease often result in treatment failure; in addition, the NB patients in either intermediate- or high-risk group present with prognostic heterogeneity. It is therefore important to identify more useful cancer biomarkers which allow for subgrouping NB patients as homogeneously as possible in terms of biology and outcome. Glycan-based therapeutics has been applied to cancers from breast, lung, gastrointestinal system, melanomas, and lymphomas but rarely to NBs [12–19]. In NB, disialoganglioside (GD2; a surface glycolipid synthesized by GD2 synthase) is uniformly expressed by virtually all neuroblasts and facilitates the attachment of NB cells to ECM [20]. This feature makes GD2 a potential molecular marker for residual disease detection and a target for immunotherapy. The success of anti-GD2 antibodies suggests that glycan-based therapies may be effective in patients with high-risk NB. Researchers have also envisaged the possibility of using protein glycosylation-based therapies to treat NB.

This review summarizes our understanding of cancer glycobiology and focuses on how protein glycosylation affects the cellular behavior and treatment outcomes of various cancers, especially NB. The effects exerted by glycosyltransferases, tumor cell-cell, and tumor cell-ECM interactions are also elucidated. Finally, this review discusses the advances in glycan-based therapies that have been utilized for a variety of cancers, such as glycosyltransferase inhibitors, glycomimetics, and glycan-based vaccines/immunotherapies. It is anticipated that NB-associated glycoforms regulated by genetic and epigenetic machinery will provide information with which novel therapeutic targets can be identified, and new therapies can be developed.

## Protein glycosylation in normal and malignant cells

### *N*- and *O*-glycosylation of proteins

Glycans are expressed in several types of glycoconjugates, namely glycoproteins, glycosphingolipids, proteoglycans/glycosaminoglycans (GAGs), and glycosylphosphatidylinositol (GPI)-linked proteins. During protein synthesis, glycans assure correct folding in the endoplasmic reticulum (ER) and are involved in trafficking of newly synthesized proteins [21]. They also protect proteins from degradation inside or outside the cell by means of interfering with proteolysis. Many glycans act as receptors for bacteria, viruses, and other pathogens. The cell-surface sugar structures allow the immune cells to differentiate self/normal cells from non-self/abnormal cells. They are also involved in cell-cell and cell-matrix interactions which are associated with cancer-cell invasion to the surrounding tissue or extravasation to form metastatic lesions [22].

**Fig. 1:**
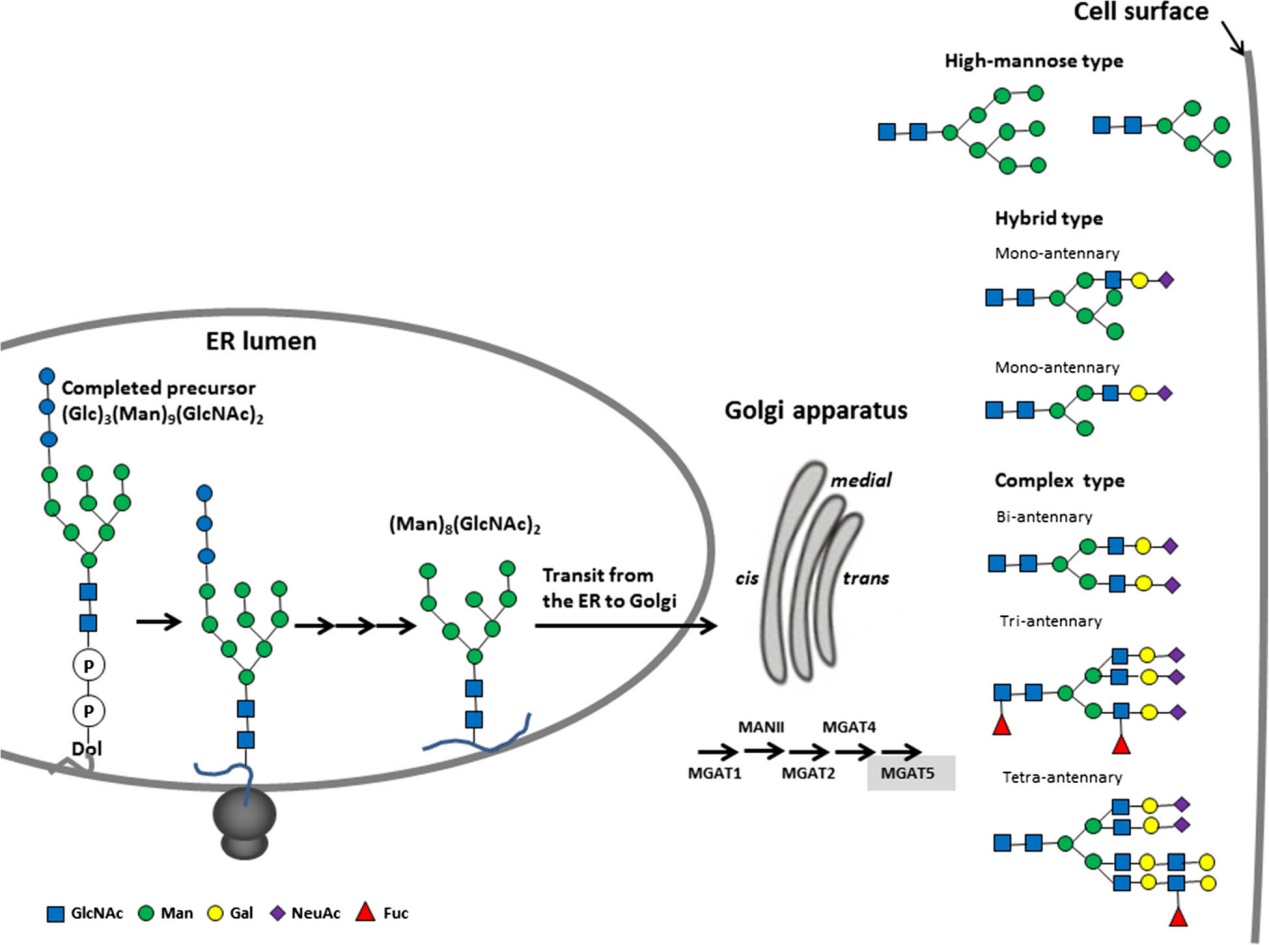
An overview of the process of *N*-glycosylation. Glycosyltransferases involved in the synthesis are indicated. Additional modifications exist (not shown). *Dol* dolichopyrophosphate, *MGAT* β1,6-*N*-acetylglucosaminyltransferase, *MANII* mannosidase II, *GlcNAc N*-acetylglucosamine, *Man* mannose, *Gal* galactose, *NeuAc N*-acetylneuraminic acid, *Fuc* fucose. Glycosyltransferases shown in Table 1 are highlighted, except B4GALNT3

**Fig. 2:**
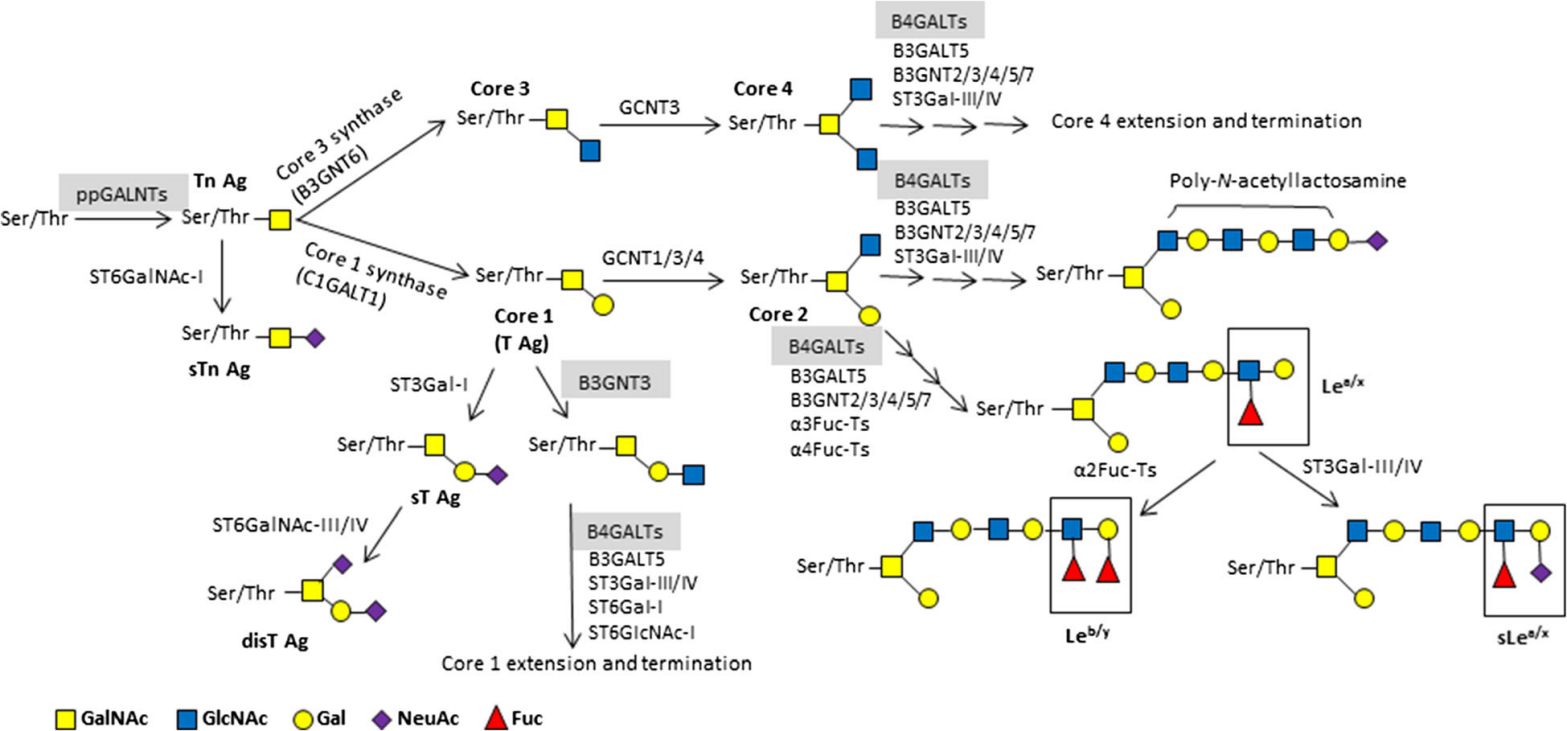
Biosynthetic pathways of mucin-type *O*-glycans. Glycosyltransferases involved in the synthesis are indicated. Additional modifications exist (not shown). *B3GALT5* β1,3-galactosyltransferase 5, *B3GNT* β1,3-*N*-acetylglucosaminyltransferase; B4GALTs, β1,4-galactosyltransferases, *C1GALT1*, core 1 β1,3-galactosyltransferase, *Fuc-T* fucosyltransferase, *GCNT* β1,6-*N*-acetylglucosaminyltransferase, *ST3Gal* Gal: α2,3-sialyltransferase, *ST6Gal-I* Gal: α2,6-sialyltransferase-I, *ST6GalNAc* GalNAc: α2,6-sialyltransferase, *ST6GlcNAc-I* GlcNAc: α2,6-sialyltransferase-I, *ppGALNTs* UDPGalNAc-polypeptide *N*-acetylgalactosaminyltransferases, *GalNAc N*-acetylgalactosamine, *GlcNAc N*-acetylglucosamine, *Gal* galactose, *NeuAc N*-acetylneuraminic acid, *Fuc* fucose. Glycosyltransferases shown in Table 1 are highlighted, except B4GALNT3

There are two major types of protein glycosylation in mammalian cells, namely *N*-linked and *O*-linked. Both types often coexist in the same protein. The synthesis of *N*-glycans is initiated in the ER by transfer of a preformed lipid (dolichopyrophosphate)-linked oligosaccharide precursor containing three glucoses, nine mannoses, and two *N*-acetylglucosamines, written as (Glc)_3_(Man)_9_(GlcNAc)_2_, to asparagine of nascent proteins. Subsequent processing occurs in the ER for protein folding, including cycles of glucose removal and addition. *N*-glycan chains can be further diversified in the Golgi apparatus as well [23]. *N*-glycans can be divided into three types according to the sugar moiety structures: high-mannose type, hybrid type, and complex type (Fig. 1). *O*-glycans are synthesized in the ER, Golgi apparatus, or cytosol by stepwise enzyme transfer of monosaccharides without the need of dolichol carrier. The frequency of *O*-glycosylation on many glycoproteins is high, especially on secreted or membrane-bound mucins, which are rich in serine and threonine. Mucin-type *O*-glycosylation which is the most common type of *O*-glycosylation in mammals is highly conserved in the evolutional course of many species; it is initiated by the transfer of *N*-acetylgalactosamine (GalNAc) to a serine or threonine residue, thereby forming the Thomsen-nouvelle antigen (Tn Ag) (Fig. 2) [24]. This reaction is catalyzed by a family of polypeptide GalNAc transferases (GALNTs) that consists of at least 20 members in humans, namely GALNT1 to 20 [25]. T synthase (or core 1 β1,3-galactosyltransferase (C1GALT1)) galactosylates Tn to form the core 1 (Galβ1 → 3GalNAc, T antigen) [26]. Core 2 is formed by adding a branching GlcNAc to core 1 by core 2 β1,6-*N*-acetylglucosaminyltransferases (GCNTs 1, 3, and 4) [27]. Alternatively to the core 1 structure formation, GlcNAc instead of Gal can be transferred in a β1–3 linkage forming the core 3 structure by β1,3-*N*-acetylglucosaminyltransferase 6 (B3GNT6) [28]. Core 3 may serve as an acceptor substrate for the core 4 enzyme, GCNT3, which adds GlcNAc in a β1-6 linkage to GalNAc (Fig. 2). Core structures 5–8 have an extremely restricted occurrence, and core 7 has not been found in humans [29]. Other non-mucin-type *O*-glycosylations are not further discussed in this review.

### Lectins (glycan-binding proteins)

Three main types of lectins, namely siglecs, galectins, and selectins, are glycan-binding proteins (GBPs) that are highly specific for sugar moieties. In healthy organisms, various endogenous lectins are associated with fundamental processes such as cell-cell recognition, cell adhesion and motility, and pathogen-host recognition. Many lectins are expressed on the surface of immune cells and endothelial cells or exist as ECM proteins and soluble adhesion molecules [1]. Siglecs are sialic acid-binding immunoglobulin-like lectins, which are expressed on specific subpopulations of hematopoietic cells such as macrophages, natural killer cells, and B cells. The binding of siglecs to tumor-derived glycans may exert various immune activities leading to anti-tumor immunity or tumor escape of immune surveillance [5]. The galectin family consists of 15 members, which are expressed by various cell types including epithelial and immune cells. Galectins belong to soluble (different from the membrane-bound nature for siglecs and selectins) immunomodulatory lectins and bind to galactose that is either β1,3 or β1,4-linked to *N*-acetylglucosamine at the cell surface, forming lattices that fine-tune the dynamics of receptor-ligand interactions. Accumulating evidence indicates that cancer-associated galectins facilitate invasive phenotype of tumor cells [30]. The selectin family consists of L-, E-, and P-selectin, which share ~50 % sequence homology in the C-type lectin domain. Their ligands typically consist of glycans capped with sialic acid, fucose, and sulfate. Selectins normally mediate adhesion of platelets (which express P-selectins), homing and development of leukocytes/lymphocytes (which express L-selectins), and recruitment of immune cells in response to inflammation (E- and P-selectins). The interactions between lectins and tumor cell-derived glycans alter tumor cell-cell interaction and cell-ECM adhesion, thereby facilitating tumor progression/dissemination [1].

### Altered protein glycosylation in cancers

**Fig. 3:**
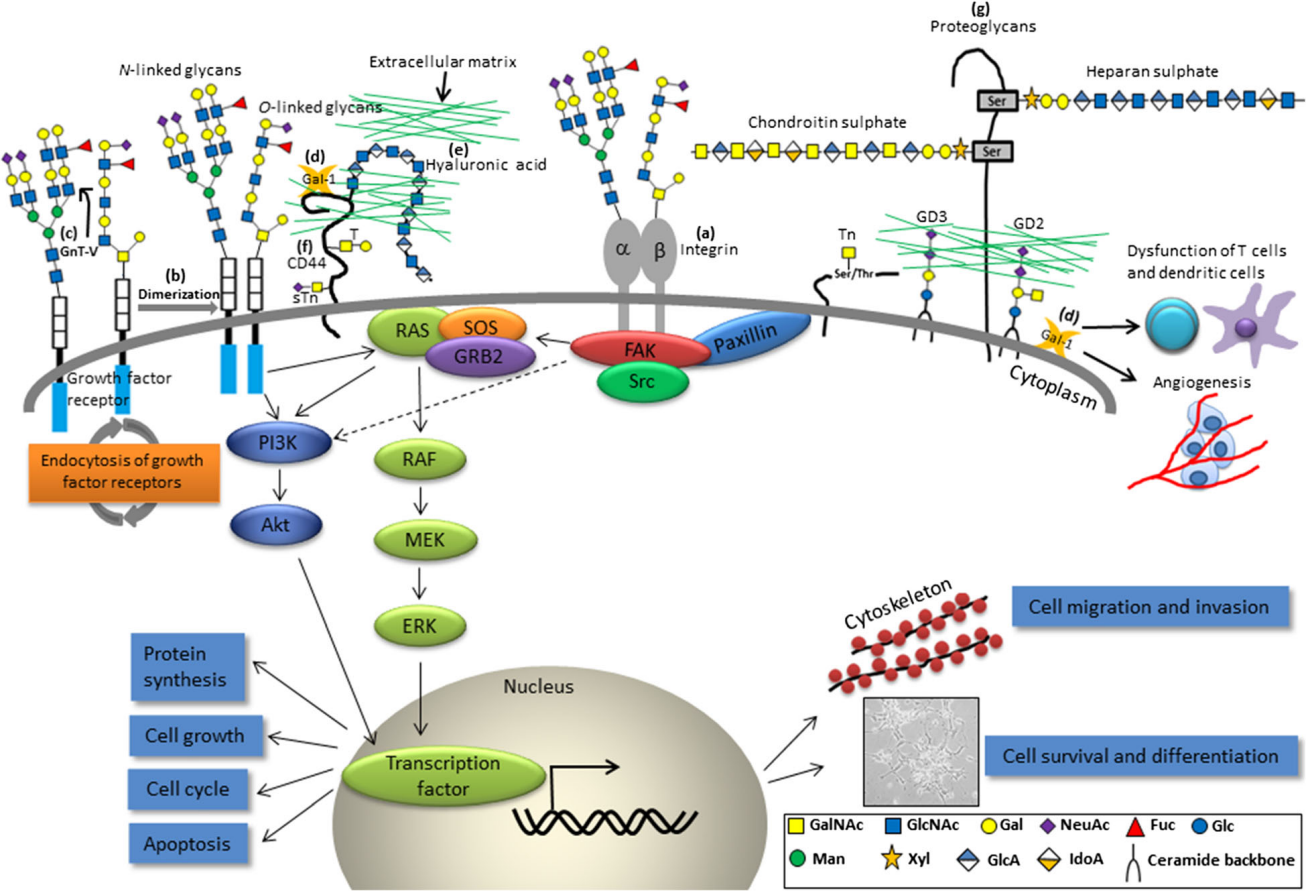
Altered glycans and related pathophysiological events involved in NB progression. **a** β1,4-*N*-acetylgalactosaminyltransferase 3 (B4GALNT3) and β1,4-galactosyltransferase 3 (B4GALT3) exhibit differential effects on malignant phenotypes by modification of β1 integrin in NB cells; **b** *N*-acetylgalactosaminyltransferase 2 (GALNT2) modifies *O*-glycans on IGF-1R, thereby suppressing IGF-1-induced IGF-1R dimerization and downstream signaling; **c** *N*-acetylglucosaminyltransferase V (GnT-V) modulates the sensitivity of NB to apoptosis; **d** Gal-1 promotes attachment of NB cells to the extracellular matrix (ECM) and endothelial cells through binding to CD44. Besides, Gal-1 may dampen the function of T cells and dendritic cells as well. Glycosaminoglycans present as **e** free polysaccharides (hyaluronic acid), a major counterreceptor for **f** CD44, or **g** as part of proteoglycans (heparan sulfate and chondroitin sulfate). *GalNAc N*-acetylgalactosamine, *GlcNAc N*-acetylglucosamine, *Gal* galactose, *NeuAc*, *N*-acetylneuraminic acid, *Fuc* fucose, *Glc* glucose, *Man* mannose, *Xyl* xylose, *GlcA* glucuronic acid, *IdoA* iduronic acid

Altered glycosylation of membrane-bound (such as cytokine or growth factor receptors, integrins, and cadherins) and secreted glycoproteins is associated with various cancers [31, 32]. Ogata et al. found that malignant cells are more enriched in highly branched complex-type *N*-linked sugar chains than their normal counterparts [33]. Many subsequent studies confirmed that an increase of β1–6 branching of the complex-type *N*-linked sugar chains is associated with cancer growth and metastasis. β1,6-*N*-acetylglucosaminyltransferase V (GnT-V; MGAT5) is one of the most relevant glycosyltransferases associated with cancer migration, invasion, and metastasis (Fig. 3). This enzyme is responsible for adding GlcNAc in a β1,6-linkage, initiating the fourth branch in a sequential pathway to tetraantennary *N*-glycans (Figs. 1 and 3) [34]. In human breast and colorectal cancers, the expression of β1–6 branched oligosaccharides regulated by GnT-V can serve as a marker for tumor progression, metastasis, and poor prognosis [35, 36]. However, GnT-V may exhibit opposite effects on other neoplasms. For example, GnT-V expression predicts a favorable prognosis and treatment outcome in lung cancers and NB [37, 38]. On the other hand, GnT-III catalyzes the attachment of a GlcNAc to a core mannose of *N*-glycan via a β1,4-linkage to form the bisecting GlcNAc structure and has been proposed to antagonize GnT-V, thereby contributing to the suppression of cancer metastasis [39].

Many mucin-type *O*-glycosyltransferases have been assigned biological functions, and aberrant expression of these enzymes is associated with human diseases. For example, the expression of *N*-acetylgalactosaminyltransferase (GALNT) 3 is a potential diagnostic and prognostic marker for lung [40] and pancreatic [41] cancers. GALNT6 can glycosylate and stabilize oncoprotein mucin 1 (MUC1), thereby contributing to mammary carcinogenesis via disruption of cell adhesion molecules (β-catenin and E-cadherin). The same research team also found that GALNT6-fibronectin pathway is also a critical component for breast cancer development and progression [42, 43]. GALNT14 modulates death-receptor *O*-glycosylation in pancreatic carcinoma, non-small-cell lung carcinoma, and melanoma cells, and may therefore serve as a predictive biomarker for Apo2L/tumor necrosis factor-related apoptosis-inducing ligand-based cancer therapy [44].

Increased expression of shorter *O*-glycan structures such as Thomsen-Friedenreich (T), Tn, sialyl-Tn (sTn), and common tumor-cell epitopes such as sialyl-Lewis A (sLe^a^) and sialyl-Lewis X (sLe^x^) has been observed in a number of carcinomas of several organs (such lung, colon, stomach, and pancreas) and is associated with malignant transformation, cancer growth, and metastatic ability [45–49]. Impaired function of glycosyltransferases responsible for the synthesis of core structures used as substrates of chain elongation and/or overexpression of sialyltransferases (such as sialyltransferases ST6GalNAc-I and ST3Gal-I) responsible for the synthesis of sTn and sT antigens may result in increased expression of incomplete glycan structures (Fig. 2) [50, 51]. The chaperone protein *Cosmc* is responsible for folding and stability of β1,3-galactosyltransferase (T synthase). Mutation in *Cosmc* chaperone leads to increased Tn and sTn expression in colon carcinoma and melanoma cells [52]. Mucins contain both *O*-linked and *N*-linked oligosaccharides and are major carriers of these cancer-associated carbohydrates. Multiple mucin domains differentially interact and regulate different components of the tumor microenvironment [53].

### Altered lectin-glycan interactions in cancers

The functional role of tumor-associated glycans has been noted to be dependent on lectin binding. Miyazaki et al. reported that non-malignant colon epithelial cells express di-sLe^a^ and 6-sulfo sLe^x^ which serve as ligands for siglec-7 and siglec-9 on resident macrophages in the colonic lamina propriae. Expression of di-sLe^a^ and 6-sulfo sLe^x^ was decreased during malignant transformation, and was replaced by increased expression of sLe^a^ and sLe^x^ which have no siglec ligand activity. Meanwhile, they found that normal glycans of epithelial cells exert a suppressive effect on cyclooxygenase-2 expression by resident macrophages, thus maintaining immunological homeostasis in colonic mucosal membranes, whereas loss of immunosuppressive glycans by impaired glycosylation during colonic carcinogenesis enhances inflammatory damage of the colonic mucosa [5]. Galectins, another type of lectins, are versatile modulators of cancer growth and metastasis. Tumor-derived galectin-1 (Gal-1) induces tumor angiogenesis and promotion of immunosuppression by T cell apoptosis in several types of cancers, including melanomas, NB, lung cancers, and pancreatic carcinomas, therefore correlating with tumor aggressiveness and metastasis [3, 4]. Expression of the α2,6-sialyltransferase-1 (ST6Gal-I) specifically resulted in increased sialylation of *N*-glycans on CD45, a receptor tyrosine phosphatase of T cell receptor for Gal-1, thereby inhibiting Gal-1 binding activity and Gal-1-induced T cell death [54]. In addition, Gal-1 might also be involved in alteration of tumor cell-cell and cell-matrix interactions and formation of platelet-cancercell complexes [1]. *N*-glycans are the major ligands for galectin-3 at the cell surface [55]. Oncogenesis increases lectin-glycoprotein lattice by up-regulating galectin-3 gene expression and higher-affinity *N*-glycan ligands, which in turn enhances the availability of tyrosine kinase receptors for epidermal growth factor (EGF), transforming growth factor (TGF)-β, insulin-like growth factor (IGF) and platelet-derived growth factor (PDGF) in a ligand-sensitive state [56]. For all three selectins, the minimal recognition epitopes are sLe^x/a^ (Fig. 2) which are present on blood cells, certain vascular endothelial cells, and glycoconjugates of the tumor-cell surface. The most common mucins carrying selectin ligands that are associated with cancer dissemination are MUC1, MUC2, MUC4, and MUC16 [57–59]. Besides, other selectin ligands carriers on tumor cells include P-selectin glycoprotein ligand-1, CD24, CD44 (which carries T and sTn antigens) (Fig. 3) [60], death-receptor 3, E-selectin glycoprotein ligand-1 [61]. Selectin-mediated interactions facilitate metastatic seeding by increased vascular permeability, forming aggregates with platelets and leukocytes, and lodgment in the small vessels of distant organs [62].

## The impacts of altered protein glycosylation on NB

### Altered *N*- and *O*-glycans in NB

*N*-linked glycosylation is a highly regulated post-translational modification, which is associated with many biological processes. It was found that the expression of intercellular adhesion molecule-2 (ICAM-2) suppresses tumor dissemination in a murine model of metastatic NB. Reduced *N*-glycosylated ICAM-2 by site-directed mutagenesis resulted in an attenuated ability to suppress metastasis of NB cells [63, 64]. Anaplastic lymphoma kinase (ALK) protein expression is up-regulated significantly in advanced/metastatic NB compared with localized NB, regardless the presence of mutated or wild-type ALK [65, 66]. Inhibition of *N*-linked glycosylation by tunicamycin affects ALK phosphorylation and disrupts downstream pro-survival signaling *in vitro*, indicating that inhibition of this post-translational modification may be a promising therapeutic approach [67]. However, as tunicamycin is not a likely candidate for clinical use, future studies will assess whether the efficacy in inhibiting ALK activity might be enhanced by the combination of ALK specific small molecules [68, 69] and *N*-linked glycosylation inhibitors.

While altered protein glycosylation is a hallmark of carcinomas, which express truncated *O*-glycans or sialylated versions of the normal counterparts at the cell surface (such as Tn, T, and sTn antigens), evidence suggests that mucin-type *O*-glycosyltransferases play an important role in NB biology, which is discussed further in the next section.

### The role of glycosyltransferases in NB

Because aberrant expression of *N*-glycans and short *O*-glycans has been detected in human NB cells, and their expression levels are regulated by associated glycosyltransferases [70], many research groups have been looking for potential glycosyltransferases as markers for residual disease detection, risk group assignment, and the prognostic factor. The authors found that GnT-V expression predicted a favorable prognosis and treatment outcome in NB. Additionally, GnT-V knockdown in NB cells resulted in a decrease in retinoic acid-induced apoptosis accompanied by morphological changes [38]. Using oligomicroarray transcriptome analysis between primary tumor versus bone marrow metastatic cell lines, Berois et al. reported that GALNT13 may serve as an informative marker for the molecular diagnosis of bone marrow involvement and the follow-up of minimal residual disease (MRD) in NB patients [71]. By contrast, GALNT9 was expressed in neuroblasts derived from the primary tumor in the IGR-N-91 NB model [72] but not in those derived from metastatic bone marrow and may serve as a prognostic marker for better clinical outcome in NB patients [73]. Over the past few years, our team had studied a series of glycosyltransferases which were reported to have prognostic impacts on NB in the public microarray datasets of Oncogenomics (https://pob.abcc.ncifcrf.gov/cgi-bin/JK). The expression status, clinical relevance, and functional role of these glycosyltransferases in NB as well as other cancers are discussed as follows.

#### β1,3-*N*-acetylglucosaminyltransferase 3

β1,3-*N*-acetylglucosaminyltransferase 3 (B3GNT3) is responsible for adding GlcNAc to core 1 (T antigen: T Ag) in a β1,3-linkage, forming extended core 1 oligosaccharides (Fig. 2). *B3GNT3* belongs to the β3GlcNAcT gene family, which consists of at least eight different β3GlcNAcTs [74]. B3GNT3 was identified for the first time to express in the high endothelial venules (HEVs) of secondary lymphoid organs; it contributes to the synthesis of HEV-borne L-selectin ligands and the lymphocyte homing [75]. B3GNT3 is also expressed in lymphocytes and neutrophils, involving in the biosynthesis of the backbone structure of sLe^x/a^, which plays a critical role in E-selectin adhesion [75, 76]. Genetic variation in *B3GNT3* gene was correlated with the risk of non-Hodgkin lymphoma [76] and the CA19-9 plasma concentration (e.g., detection of sLe^a^ epitope) [77]. However, our research team demonstrated that B3GNT3 expression examined using immunohistochemistry (IHC) correlates positively with the histological grade of differentiation as well as favorable Shimada histology and is an independent prognostic factor of better survival outcome for NB. Cell line experiments demonstrated that B3GNT3 expression decreases core 1 (T antigen) as well as malignant phenotypes including migration and invasion (Table 1) [78].

**Table 1:** Glycosyltransferases as prognostic markers with differential effects on neuroblastoma and other cancers

Enzymes	Glycosylation involved	Target proteins and associated signaling pathways	Clinical significance
β1,3-*N*-acetylglucosaminyltransferase 3 (B3GNT3)	*O*-glycosylation	B3GNT3 inhibits NB cell migration and invasion by suppression of FAK, Akt, and ERK activation.	Predicts good prognosis in NB [78].
β1,4-*N*-acetylgalactosaminyltransferase 3 (B4GALNT3)	*N*- and *O*-glycosylation	B4GALNT3 inhibits NB cell migration and invasion by modifying β1 integrin with LacdiNAc, thereby suppresses the activation of Akt and ERK signaling pathways. B4GALNT3 enhances the stemness, migration, and invasion by modifying primarily *N*-glycans with LacdiNAc on EGFR and downstream signaling in CRC cells.	Predicts good prognosis in NB [81]. Predicts poor prognosis in CRC [83].
β1,4-galactosyltransferase 3 (B4GALT3)	*N*- and *O*-glycosylation	B4GALT3 increases NB cell migration and invasion by modifying lactosamine structures of β1 integrin, delaying the degradation of β1 integrin, and enhancing its downstream signaling. B4GALT3 suppresses CRC cell migration and invasion by inhibiting β1 integrin activation through altering the poly-*N*-acetyllactosamine expression on *N*-glycans of β1 integrin.	Predicts poor prognosis in NB [86]. Predicts good prognosis in CRC [87].
*N*-acetylgalactosaminyltransferase 2 (GALNT2)	*O*-glycosylation	GALNT2 regulates NB cell growth, migration, and invasion by modifying *O*-glycans on IGF-1R, thereby suppressing IGF-1-induced IGF-1R dimerization and downstream signaling. GALNT2 inhibites HCC cell proliferation, migration, and invasion by modifying *O*-glycans on EGFR, thereby suppressing EGF-induced endocytosis of EGFR and downstream signaling. GALNT2 enhances OSCC cell migration and invasion by modifying *O*-glycosylation and activity of EGFR.	Predicts good prognosis in NB [90]. Predicts good prognosis in HCC [91]. Predicts poor prognosis OSCC [92].
*N*-acetylglucosaminyltransferase V (GnT-V; MGAT5)	*N*-glycosylation	GnT-V knockdown results in a decrease in the susceptibility to cell apoptosis induced by retinoic acid in NB cells accompanied by morphological change	Predicts good prognosis in NB [38].

*FAK* focal adhesion kinase, *ERK* extracellular signal kinase, *LacdiNAc* the structure of GalNAcβ1,4GlcNAc

#### β1,4-*N*-acetylgalactosaminyltransferase 3

β1,4-*N*-acetylgalactosaminyltransferase 3 (B4GALNT3) has been cloned, and its transcript is highly expressed in stomach, colon, and testis [79]. This enzyme can transfer GalNAc to any nonreducing terminal GlcNAc-β* in vitro*, resulting in the synthesis of GalNAcβ1,4GlcNAc (LacdiNAc or LDN). This special terminal β1,4GalNAc structure is found in certain glycoproteins and glycohormones; one of them is the sorting protein-related receptor SorLA/LR11 which shuttles between the plasma membrane, endosomes, and the Golgi. SorLA/LR11, highly expressed by neurons in the central and peripheral nervous systems, bears *N*-linked oligosaccharides modified with terminal β1,4-linked GalNAc-4-SO_4_ that can be synthesized by B4GALNT3 in CHO cells [80]. By using IHC analysis to examine the B4GALNT3 expression in NB tumors, we found that B4GALNT3 expression correlates positively with the differentiation status of NB and early clinical stage (INSS stage 1, 2, and 4S) and may predict a favorable prognosis independently. Cell line experiments demonstrated that B4GALNT3 suppresses NB cell proliferation, migration, and invasion primarily by increasing LacdiNAc modification of β1 integrin, thereby inhibiting the downstream signaling of β1 integrin (Table 1) [81]. However, in human colon cancers, our research team previously presented that *B4GALNT3* messenger RNA (mRNA) is frequently up-regulated in primary colon cancer tumors compared with their normal counterparts. B4GALNT3 overexpression significantly promotes malignant behaviors of colon cancer cells both *in vitro* and *in vivo* through enhanced mitogen-activated protein kinase (MAPK) signaling pathways [82]. We later found that B4GALNT3 expression examined using IHC staining correlates positively with advanced American Joint Committee on Cancer stages, high metastasis rates, and poor survival in colorectal cancer patients. Moreover, cell line experiments revealed that B4GALNT3 expression regulates cancer stemness and the invasive properties of colon cancer cells through modifying epidermal growth factor receptor (EGFR) glycosylation and downstream signaling (Table 1) [83].

#### β1,4-galactosyltransferase 3

Another interesting glycosyltransferase, β1,4-galactosyltransferase 3 (B4GALT3), is a member of the B4GALT family composed of seven isoenzymes and is responsible for transferring galactose from UDP-Gal to GlcNAc-terminated oligosaccharides on *N*-glycans, *O*-glycans, glycolipids, or GAG chains to form poly-*N*-acetyllactosamines (Fig. 2) [84]. B4GALT3 has been noted to have higher expression levels in fetal brains than in adult brains [85]. Our research team found that positive B4GALT3 expression examined by IHC staining in NB tumor tissues correlates negatively with the histological grade of differentiation and early clinical stage and is an unfavorable prognostic factor independent of other factors for NB patients. Moreover, B4GALT3 increases poly-*N*-acetyllactosamine levels on the mature form of β1-integrin, which delays β1-integrin degradation and enhances its downstream signaling, thereby increasing NB cell migration and invasion (Table 1) [86]. Our team later reported that the expression level of B4GALT3 in colorectal cancer (CRC) patients negatively correlates with poorly differentiated histology, advanced stages, and metastasis. Cell line experiments revealed that B4GALT3 overexpression inhibits CRC cell malignant phenotypes by decreasing the synthesis of poly-*N*-acetyllactosamines on *N*-glycans of β1-integrin, which in turn suppresses its downstream signaling related to cell attachment to ECM, cell migration, and invasion (Table 1) [87].

#### *N*-acetylgalactosaminyltransferase 2

*N*-acetylgalactosaminyltransferase 2 (GALNT2), a member of the GALNT family responsible for initiation of mucin-type *O*-glycosylation (Fig. 2), has been found to express differentially in nervous tissues during mouse embryogenesis [88]. The expression of GALNT2 also regulates migration and invasion of human glioma cells* in vitro* [89]. In NB, we found that increased GALNT2 expression examined using IHC in primary tumor tissues correlates well with the histological grade of differentiation and early clinical stage and may serve as an independent prognostic factor for better survival of NB patients. *In vitro* and *in vivo* experiments using overexpression and knockdown revealed that the expression of GALNT2 suppresses IGF-1-induced cell growth, migration, and invasion of NB cells by modifying *O*-glycans on IGF-1R, which suppresses IGF-1-triggered IGF-1R dimerization and subsequent downstream signaling events (Table 1) [90]. In hepatocellular carcinoma (HCC), we observed that *GALNT2* mRNA expression exhibits significant down-regulation in HCC tissues compared with normal counterparts. We also found that GALNT2 expression suppresses cell malignant phenotypes by modulating the *O*-glycosylation of EGFR, which inhibits EGF-induced endocytosis and colocalization of EGFR and its downstream signaling [91]. In oral squamous cell carcinoma (OSCC), although GALNT2 expression can also modify the *O*-glycosylation of EGFR, it can facilitate the activation and downstream signaling of EGFR, thereby enhancing OSCC cell migration and invasion (Table 1) [92].

#### Glycosyltransferases may serve as biomarkers for NB

From the series of studies above, it is postulated that different cell types possess differential expression patterns (repertoire) of isoenzymes, which may result in different glycosylation sites or densities of cell-surface receptors [93]. GALNT2, for example, may add GalNAc to different *O*-glycosites on EGFR in HCC and OSCC cells, which is determined by the repertoire of GALNTs in respective cells. Therefore, EGFR glycoforms modulated differentially by GALNT2 in these cells may present with opposite cellular properties, including cell proliferation, growth, oncogenesis, and metastasis. Moreover, differential sialylation patterns of shorter glycans (such as sTn, sLe^a/x^, or di-sLe^a/x^) regulated by sialyltransferases are also attributed to specific glycoforms at the cell surface of these cancers.

Taken together, these enzymes could be potential candidates for a panel of tumor markers for MRD detection or treatment outcome of NB patients, yet the significance of individual enzymes and more detailed molecular mechanisms need further investigation. They will help to define and develop personalized treatment for patients with NB. Moreover, they may provide an alternative approach to cancer therapy by means of modulating cancer-specific glycosylation.

### The roles of Gal-1 and CD44 in NB microenvironment

Soluble Gal-1 has been found to be secreted by different NB cell lines and was identified as a major mediator of TrkB-mediated NB aggressiveness [6]. Additionally, NB-derived Gal-1 may dampen the function of T cells and dendritic cells [94]. Gal-1 may also mediate attachment of cancer cells to the ECM and endothelial cells through binding to CD44 (Fig. 3) [95].

CD44, a complex transmembrane glycoprotein, was found to be closely associated with the development of various solid tumors in terms of cancer stemness and epithelial-mesenchymal transition (EMT) [60]. However, CD44 is generally downregulated in human NB cells [96]. Unlike other cancers, the absence of CD44 expression indicates aggressiveness and poor clinical outcome in NB [97]. One of the CD44 isoforms, CD44v6, contains sequences encoded by variant exon v6. CD44v6 serves as the carrier of T and sTn antigens in colon cancer cells and has been linked to metastatic spreading of a number of malignancies [60]. In NB, the induction of CD44V isoform (especially CD44v6) expression by 12-*O*-tetradecanoyl phorbol-13-acetate (TPA), IGF-1, and PDGF was correlated with an increased cellular binding to hyaluronic acid (a major counterreceptor for CD44; free form of GAGs in ECM) by phosphoinositide 3-kinase (PI3K)/protein kinase C (PKC) pathways, indicating the impact of glycosylation status and local distributions of the molecule on the changes in NB cell properties (Fig. 3) [98]. The interactions between Gal-1, CD44, and other associated molecules have made them become interesting therapeutic targets and/or prognostic factors for patients with NB.

### Disialoganglioside expression and therapeutic applications in NB

In normal human tissues, GD2 is a surface glycolipid antigen expressed on neurons, skin melanocytes, and peripheral sensory nerve fibers. In NB, GD2 (synthesized by the GD2 synthase) is uniformly expressed by virtually all neuroblasts and is involved in the attachment of NB cells to ECM proteins (Fig. 3) [20]. This feature makes GD2 potentially suitable for a molecular marker for residual disease (but not a prognostic marker) and a target for immunotherapy. Although the use of isotretinoin (13-*cis*-retinoid acid) has been incorporated into the standard treatment as a biotherapy for high-risk NB patients, monoclonal antibodies directed against NB-specific antigens such as GD2 may provide another efficacious approach to eliminate residual NB cells by complement-dependent cytotoxicity (CDC) and antibody-dependent cell-mediated cytotoxicity (ADCC). There are four major anti-GD2 monoclonal antibodies (mAbs) (3F8, hu3F8, ch14.18, and hu14.18) being extensively tested in clinical settings [12]. A phase III randomized study reported by Yu et al. showed that adding immunotherapy (ch14.18 in combination with granulocyte-macrophage colony-stimulating factor (GM-CSF) and interleukin (IL)-2 to enhance the ADCC) to isotretinoin therapy, as compared with the use of isotretinoin alone, improved the survival of children with high-risk NB in remission after myeloablative therapy and stem-cell rescue [99]. A recent study even suggests that autologous stem-cell transplantation may not be needed to improve outcome when anti-GD2 immunotherapy is used for consolidation after dose-intensive conventional chemotherapy [100]. Further clinical investigations of several novel combinatorial immunotherapies are underway and will provide new hope for infants and children with NB.

## Conclusions

### Glycan-based therapeutics in cancers

The presence of glycans on various biomolecules shows their physiological and pathological importance; therefore, glycan-based therapeutics has been developed for treatment of cancers and other diseases, such as glycosyltransferase inhibitors, glycomimetics, glycan/glycopeptide vaccines, antibody therapies, and antibody-based immunotherapies. Sialic acid and fucose are the common glycan structures on the terminal branches of *N*- and *O*-glycans of glycoproteins and are involved in cancer progression and metastasis. It has been reported that the cell-permeable, fluorinated analogs of fucose and sialic acid can be used as inhibitors of both fucosyltransferases and sialyltransferases and drastically reduced sLe^x^ expression on myeloid cells, resulting in loss of binding to selectins and impaired leukocyte rolling [101]. Several other inhibitors that target sialyltransferases have been developed, such as soyasaponin-I (selectively inhibits α2,3-sialyltransferases activity) and AL10 (inhibits α2,3- and α2,6-sialyltransferases activity), for treatment of metastatic breast and lung cancer cells, respectively [13, 14]. Glycomimetic drugs such as acetylated or/and fluorinated derivatives of glycans (4F-GalNAc and 4F-GlcNAc) were used as decoys to disrupt the biosynthesis of natural ligands for selectins, which blocked selectin interaction with the endogenous glycans and mediated cellular adhesion [102]. These studies may pave the way for the broader use of these glycosyltransferase inhibitors or glycomimetics in NB patients. However, because all members of a given family utilize the same donor substrate (such as GlcNAc for B3GNT3, GalNAc for B4GALNT3 or GALNTs), the differential cellular expression pattern and acceptor specificity of each enzyme in NB cells need to be clarified further in the future..

Unusual glycomotifs on glycoproteins can be recognized by the immune system, but they are weak immunogens. Therefore, vaccines of this type are typically prepared by conjugating the glycan to a carrier protein to boost both humoral and cellular immune responses, such as the use of sTn-KLH vaccines in phase III clinical trials for breast cancer patients with metastatic disease [103]. Other glycan/glycopeptide vaccines have been designed and generated to elicit more robust immune responses, such as the development of synthetic vaccines consisting of a MUC1 glycopeptide along with a T helper (Th) peptide [104], a MUC1 glycopeptide along with toll-like receptor 2 (TLR2) lipopeptide ligands [105] or a MUC1 glycopeptide along with a Th peptide and a TLR2/TLR9 agonist [106]. Multivalent vaccines targeting multiple mucins have been developing against various cancers [22]. To date, advances in availability of recombinant glycosyltransferases have made it possible to synthesize cancer-associated antigens mimicking the surface of cancer cells in terms of glycosylation sites and density, which allows for specific targeting cancer cells with immunotherapeutics [22].

NB-associated glycoforms provide information with which cancer vaccines are designed and synthesized. For example, a phase I trial of a bivalent GD2-GD3 gangliosides vaccine in combination with the immunostimulant β-glucan revealed an encouraging result in high-risk NB patients [107]. Another vaccine target used for NB is *N*-glycolyl GM3 (NeuGcGM3), which is expressed in 85 % of NB cases, including those with *MYCN* amplification [108]. Racotumomab is a murine anti-idiotype vaccine that mimics NeuGcGM3 and was reported to trigger an anti-NeuGcGM3 response in adults with melanomas [15], lung cancers [16], and breast cancers [17]. A phase I study using racotumomab in children with NB and other refractory malignancies revealed that most patients elicited an immune response against racotumomab but did not show significant anti-tumor activity in most cohort patients who were heavily pre-treated with front-line therapy. Therefore, evaluation of long-term vaccination with this vaccine is underway [109].

### Perspectives on glycosylation-based therapies for NB

It is well-known that receptor kinases that mediate arrest and differentiation tend to have low multiplicity, and their dependency on *N*-glycan branching or *O*-glycosylation for surface residency is altered by oncogenesis-driven membrane remodeling. We reason that “nontransformed” dynamics can be restored by jointly manipulating protein synthesis, *N*- or *O*-glycosylation, and Golgi remodeling to change the cell-surface glycosylation pattern involved in growth and arrest [34]. Moreover, epigenetic mechanisms on glycogenes (e.g., DNA methylation, histone modifications, and non-coding RNAs [110–117]) (Table 2) associated with cell-surface glycosylation in various cancers may develop as potential targets for anti-tumor therapy in the near future.

**Table 2:** Identified glycogene/miRNA interactions in human diseases

miRNAs	Glycogene targets	Comments
miR-30b/30d	GALNT1, GALNT7	Both GALNT1 and GALNT7 are targets of miR-30b/d, which are associated with metastasis in melanoma [112].
miR-378	GALNT7	GALNT7 is a target of miR-378 and plays a critical role in osteoblast differentiation [111].
miR-122	GALNT10, FUT8	GALNT10 modulates *O*-glycosylation of EGFR in hepatitis B virus (HBV)-infected hepatoma cells. GALNT10 is a target of miR-122, whose gene transcription is activated by hepatocyte nuclear factor 4α (Hnf4α). Therefore, a regulatory pathway of Hnf4α/miR-122/GALNT10/EGFR may develop as therapeutic targets [113]. Ectopic expression of miR-122 can significantly decrease FUT8 levels, thus may play a role in the dysregulation of core fucosylation observed in liver tumors [114].
miR-27a	B4GALT3	B4GALT3 up-regulated by miR-27a contributes to the tumorigenic activities by β1-integrin pathway and might provide potential biomarkers for cervical cancer [117].
miR-148b	C1GALT1	Inhibition of miR-148b expression can reverse the lower levels of C1GALT1 typical of IgA nephropathy. Therefore, miR-148b levels may be manipulated to provide a therapeutic approach to the disease [110].
miR-199b-5p	FUT4	The cluster of differentiation carbohydrate antigen CD15, also known as FUT4, is a marker of medulloblastoma tumor-propagating cells and an additional direct target of miR-199b-5p. Therefore, the finely tuned regulation of miR-199b-5p may have a role in therapeutic application in medulloblastoma [115].
miR-34a	FUT8	Ectopic expression of miR-34a can significantly decrease FUT8 levels, thus may play a role in the dysregulation of core fucosylation observed in liver tumors [114].
miR-125b	ERManI	ERManI functions as a “gate keeper” in the Golgi complex to facilitate the retention and recycling of misfolded glycoproteins escaped from the ER. In hepatoma cells, however, ERManI regulates transformation phenotypes independent of ER-stress. ERManI knockdown by miR-125b inhibits proliferation and migration of hepatoma cells [116].

*B4GALT3* β1,4-galactosyltransferase 3, *C1GALT1* core 1 β1,3-galactosyltransferase, *FUT* fucosyltransferase, *GALNT N*-acetylgalactosaminyltransferase, *ERManI* human endoplasmic reticulum alpha-1, 2-mannosidase I

So far, almost all approved mAbs are targeting protein antigens, except anti-GD2 mAbs. Anti-GD2 antibodies have been actively tested since 1980s in various preclinical and clinical combinatorial trials for NB, and now have proved their safety and efficacy when combined with GM-CSF and IL-2 in the treatment of high-risk NB patients. An interesting fusion protein, namely hu14.18-IL2, has been generated by fusing an IL-2 moiety to hu14.18 mAb. This fusion protein has shown superior anti-tumor activity as compared with ch14.18 mAb combined with IL-2 in NB patients with nonbulky disease [118]. Building on the success of ch14.18, researchers are using T cells, engineered to express a new class of proteins known as chimeric antigen receptors (CARs), to enhance anti-tumor efficacy. Therefore, engineered human T lymphocytes expressing GD2-directed CARs (GD2-CARs) were generated. Louis et al. found that these GD2-CAR T cells can induce complete tumor responses in patients with active NB and have extended, low-level persistence associated with longer survival outcome in these patients [119].

Many approaches have been developed to improve the efficacy of therapeutic antibodies; Potelligent^®^ Technology is one of the most potent technologies for enhancing ADCC [120]. The concept of this technology originated from the discovery that reducing or eliminating fucose from the oligosaccharides on the Fc domain significantly increased FcγRIIIa binding and dramatically enhanced ADCC by ~100-fold [121]. In addition, an antibody without fucose is a natural component of human serum and therefore has a lower risk of immunogenicity. A defucosylated anti-CC chemokine receptor 4 (CCR4), mogamulizumab, not only induces ADCC against CCR4^+^ malignant T cells but also reduces CCR4^+^ regulatory T cells (Tregs) which in turn restores NK cell anti-tumor function in patients with cutaneous T cell lymphoma (CTCL) [18]. This drug was approved in Japan for CCR4-positive adult T cell leukemia/lymphoma (ATL) in 2012 [19], relapse/refractory CCR4-positive peripheral T cell lymphoma (PTCL) and CTCL in 2014. Another form of defucosylated antibody, nivolumab, is a programmed death-1 (PD-1) immune-checkpoint inhibitor. PD-1 is a key immune-checkpoint receptor expressed by activated T cells and mediates immunosuppression. Blockade of the interaction between PD-1 (on activated T cells) and PD-L1 (PD-1 ligands; expressed on tumor cells or stromal cells) can enhance T cell activity and anti-tumor activity [122]. Nivolumab is approved to be used alone or with other drugs to treat metastatic melanoma, non-small cell lung cancer, and renal cell carcinoma. Anti-GD2 mAb in combination with anti-PD-1 mAb has been added to the present treatment protocols for high-risk NB and is likely to be studied in the future [123, 124]. The mutated humanized antibody, hu14.18K322A (lysine to alanine in the CH2 region critical for complement activation) produced by YB2/0 cells, showed decreased fucosylation activity and demonstrated increased ADCC and less complement activation (which was related to hypersensitivity reactions) than ch14.18. Therefore, this antibody has the potential to be less toxic, allowing for higher maximum tolerated dose (MTD) and improved efficacy for refractory/recurrent NB patients [125].

### Where do we go from here?

Recent advances in cancer cell glycomics have expanded the armamentarium of NB therapeutic targets. However, treatment of high-risk NB is still challenging because ~40 % of patients still relapse during or after glycan-based immunotherapy following standard therapy [99]. In the last two decades, several anti-GD2 antibodies and GD2-GD3 vaccines have been developed and tested in clinical trials, resulting in various treatment responses. Development of an immune response is always a concern with genetically engineered mAbs, such as human anti-mouse antibodies (HAMA), human anti-human antibodies (HAHA), or anti-chimeric-antibodies, which may influence the level of antibody therapy and potentially contribute to the undesirable adverse effects [126, 127]. Maintaining anti-tumor antibodies over months or years is more readily achievable with vaccines than with mAbs. However, Kushner et al. postulated that fluctuations in anti-GD2 titers in vaccinated patients may represent multiple idiotypes within an idiotypic network [107]. Nevertheless, these results support the idea of using GD2-GD3 vaccine as an adjuvant therapy in patients with low disease burdens but at high risk for relapse and with target antigens highly expressed in cancerous tissues but not normal tissues, after these patients have completed the standard upfront multimodal treatments [107].

In conclusion, aberrant protein glycosylation in tumor cells and NB microenvironment provides clinicians with additional opportunities to screen for specific glycosyltransferase inhibitors, glycomimetics, and glycan-based vaccines/immunotherapies. This feature also allows the development of novel genetically and epigenetically based therapies. Further investigation of the intricate glycoforms on NB cells may lead to the identification of more useful prognostic, diagnostic, and therapeutic targets in the future.

## Abbreviations

ADCC: Antibody-dependent cell-mediated cytotoxicity
ALK: Anaplastic lymphoma kinase
ATL: Adult T cell leukemia/lymphoma
B3GNT3: β1,3-*N*-acetylglucosaminyltransferase 3
B3GNT6: β1,3-*N*-acetylglucosaminyltransferase 6
B4GALNT3: β1,4-*N*-acetylgalactosaminyltransferase 3
B4GALT3: β1,4-galactosyltransferase 3
C1GALT1: Core 1 β1,3-galactosyltransferase
CARs: Chimeric antigen receptors
CCR4: CC chemokine receptor 4
CDC: Complement-dependent cytotoxicity
CRC: Colorectal cancer
CTCL: Cutaneous T cell lymphoma
ECM: Extracellular matrix
EGF: Epidermal growth factor
EGFR: Epidermal growth factor receptor
EMT: Epithelial-mesenchymal transition
ER: Endoplasmic reticulum
GAGs: Glycosaminoglycans
Gal-1: Galectin-1
GalNAc: *N*-acetylgalactosamine
GALNT: *N*-acetylgalactosaminyltransferase
GBP: Glycan-binding protein
GCNT: β1,6-*N*-acetylglucosaminyltransferase
GD2: Disialoganglioside
GlcNAc: *N*-acetylglucosamine
GM-CSF: Granulocyte-macrophage colony-stimulating factor
GnT: β1,6-*N*-acetylglucosaminyltransferase
GPI: Glycosylphosphatidylinositol
HAHA: Human anti-human antibodies
HAMA: Human anti-mouse antibodies
HCC: Hepatocellular carcinoma
HEVs: High endothelial venules
ICAM-2: Intercellular adhesion molecule-2
IGF: Insulin-like growth factor
IHC: Immunohistochemistry
IL-2: Interleukin-2
INSS: International neuroblastoma staging system
LDN: LacdiNAc or GalNAcβ1, 4GlcNAc
mAbs: Monoclonal antibodies
MAPK: Mitogen-activated protein kinase
MGAT: β1,6-*N*-acetylglucosaminyltransferase
MRD: Minimal residual disease
MTD: Maximum tolerated dose
MUC: Mucin
MYCN: V-myc myelocytomatosis viral-related oncogene
NeuGcGM3: *N*-glycolyl GM3
OSCC: Oral squamous cell carcinoma
PD-1: Programmed death-1
PDGF: Platelet-derived growth factor
PD-L1: PD-1 ligand
PI3K: Phosphoinositide 3-kinase
PKC: Protein kinase C
PTCL: Peripheral T cell lymphoma
sLe^a^: Sialyl-Lewis A
sLe^x^: Sialyl-Lewis X
ST3Gal: Gal:2,3-sialyltransferase
ST6Gal-I: Gal: α2,6-sialyltransferase-I
ST6GalNAc: GalNAc:2,6-sialyltransferase
sTn: Sialyl-Tn
T: Thomsen-Friedenreich
TGF: Transforming growth factor
Th: T helper
TLR: Toll-like receptor
Tn: Thomsen-nouvelle
TPA: 12-*O*-tetradecanoyl phorbol-13-acetate
Tregs: Regulatory T cells
